# Editorial on Pediatric Radiology – BSR Annual Meeting 2017

**DOI:** 10.5334/jbr-btr.1443

**Published:** 2017-11-18

**Authors:** Brigitte Desprechins, Luc Breysem

**Affiliations:** 1 NDB CHU Liège, BE; 2 UZ Leuven, BE

**Figure:**
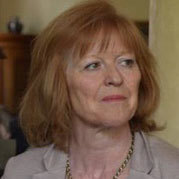
Brigitte Desprechins (Chair of the Pediatric Radiology Section of the BSR)

**Figure:**
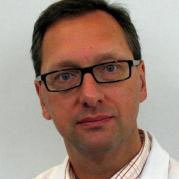
Luc Breysem (Secretary of the Pediatric Radiology Section of the BSR)

The age of the pediatric patient visiting an imaging department ranges from 24 weeks gestational age to 16 years. Recently, prenatal imaging has gained an important place in the extensive area of interest in pediatric radiology. Note that children should not be seen as little adults, as they suffer from specific pathologies that do not occur in adults. Moreover, irradiating examinations should be avoided in children as much as possible and when used, the As Low As Reasonably Achievable (ALARA) principle applies stringently.

Depending on the specific symptoms of the child and the potentially affected organ system, most questions can be answered with ultrasound (US) and that is rightly the most frequent initial imaging approach. US is non-invasive, easily accessible and applicable, and does not use ionizing radiation, remaining however operator dependant. The role of conventional radiography and computed tomography (CT) is important primarily in the field of lung and bone pathology. During the last decades however, the use of CT has substantially decreased and been replaced by magnetic resonance (MR) whenever possible. This meeting session aims to cover the most recent insights and technical evolutions in the pediatric radiology through the lectures of three experts with international recognition.

Imaging in pediatric cancer has several goals, including diagnosis, staging, monitoring disease during and after treatment and diagnosing complications of treatment. Our first speaker, Dr. Anne Smets has a special interest in pediatric oncology and her lecture is entitled: “Imaging in Pediatric Oncology in the real world”. Dr. Smets will guide us through the different imaging approaches of cancers and cancer-related pathologies, with a special emphasis on the newer techniques.

**Figure:**
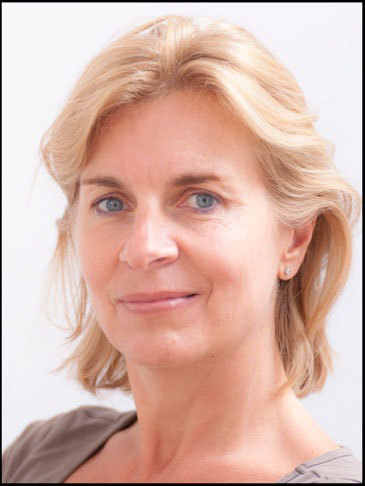
Anne Smets

*Dr. Anne Smets (Free University of Brussels (VUB) 1987) did her radiology residency with a special focus on pediatric radiology. Her first staff position was at the University Hospital in Ghent where her interest in pediatric oncology began. She continued her career at the Academic Medical Center of Amsterdam, where she has been working as a pediatric radiologist for the past 20 years. She is actively involved in pediatric cancer as chairwoman of the oncology task force of the European Society of Pediatric Radiology (ESPR) and of the radiology panel of the SIOP Renal Tumour Study Group (RTSG) and as a fellow at the International Cancer Imaging Society (ICIS). She is a regular lecturer and teacher at international conferences and courses, with pediatric oncology as her main subject*.

*She has published 34 peer-reviewed papers referenced on PubMed and is author or co-author of several book chapters. She is a reviewer for* Pediatric Radiology, European Radiology, European Journal of Radiology, British Journal of Radiology, *and* Insights into Imaging.

An optimal diagnosis of prenatal abnormalities is necessary to improve prenatal and postnatal management. For at least two decades, fetal MR has progressively established itself as the mainstay to visualize the fetus when US visualization and diagnosis is inadequate. In this respect, we invited the second speaker, Dr. Marie Cassart. Dr. Cassart is experienced in both prenatal ultrasound and MR. Her lecture is entitled “Fetal Body Imaging: When Is MRI indicated?” and provides an overview of the most commonly encountered chest, abdominal and urinary fetal pathologies in which MR helps fine-tune the diagnosis.

**Figure:**
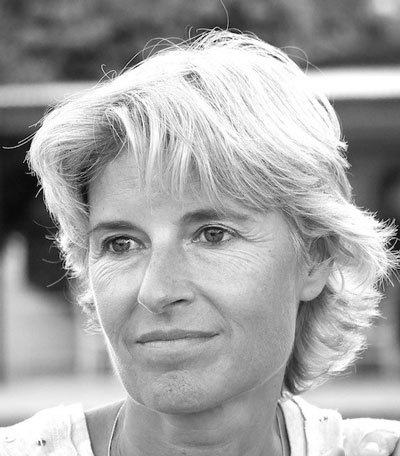
Marie Cassart

*Dr. Marie Cassart was born in Brussels. She is a pediatric radiologist specializing in fetal imaging. She gained her MD in 1991 and PhD in 2000 from the Free University of Brussels*.

*She has specialized in fetal imaging since 2000 (US, CT scanner and MRI), which represents half the time of her clinical activity. Dr. Cassart is head of the department of Perinatal Imaging in the Radiology Department of HIS Hospitals in Brussels and she is a consultant in fetal imaging in the Fetal Medicine Department at the University Hospital St Pierre in Brussels*.

*She is currently a member of many pediatric and fetal scientific societies and boards (French speaking society of Perinatal and Pediatric Imaging (SFIPP), Radiologic Group of Research in Fetal Imaging (GRRIF), etc.). She is a titular member of the SRBR and a member of the* European Radiology *editorial board*.

*Dr. Cassart has authored or co-authored around 70 scientific articles including 40 on pediatric or fetal imaging. She presented 40 international invited lectures and participated in 20 book chapters on fetal or pediatric imaging, 10 as a first author. She is co-editor-in-chief of a fetal and pediatric textbook entitled* Imagerie du foetus au nouveau-né, *published in December 2015 (Lavoisier Edition)*.

Although ultrasound is one of the most accessible and used imaging modalities for investigating children, one disadvantage is that image quality is subject to the ultrasound equipment settings and to the operator performing the ultrasound. To achieve an optimal image quality and result of the examination, it is extremely important that we know how to optimize the use of our ultrasound equipment. That’s why Prof. Dr. Michael Riccabona, an expert in the field, will provide a lecture entitled “Basics of Pediatric US Revisited: What to Consider for Providing an Excellent US Investigation, and How to Improve Image Quality”.

**Figure:**
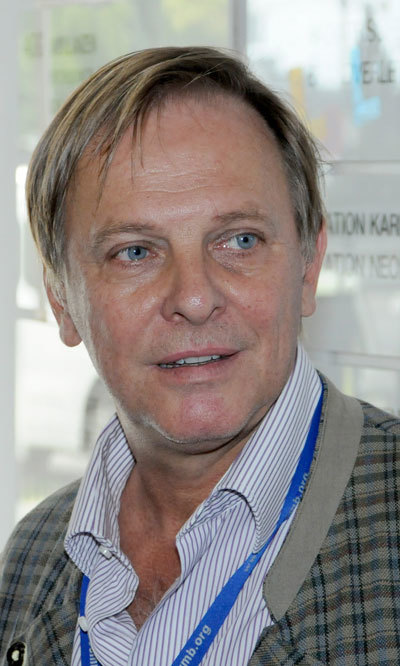
Michael Riccabona

*Michael Riccabona, Proffessor of Radiology and Pediatrics, was born in 1956 in Innsbruck, Austria. After specializing in pediatrics in the University Hospital Graz where he was in charge of pediatric radiology from 1990 to 1994, he specialized in radiology*.

*Prof. Riccabona completed several scholarships and professorships in the USA, Austria and Thailand and is member of various societies, including ESR, ESUR, ESPR, GPR, OERG, AIUM, OEGUM, DEGUM, often with a chair position. He has been committee member of numerous symposiums, congresses, meetings, courses etc. and has given lectures and workshops all over the world, especially on modern pediatric US, three-dimensional US, contrast-enhanced US, pediatric MR-urography, pediatric low-dose CT, and other aspects of modern pediatric radiology as well as uroradiology*.

*Prof. Riccabona acts as reviewer for* Eur Radiol, Eur J Radiol, Pediatr Radiol, JUM, Ultraschall im Medizin, RöFo, Urologe, Journal of Ultrasonography, der Radiologe, *etc. and is currently a member of the editorial board of the* Polish Journal of Ultrasonography *and section editor of* Pediatric Radiology.

*Prof. Riccabona has an ongoing commitment to postgraduate education for colleagues from Eastern Europe (particularly former Soviet countries, “EAR-Tutorial”, ESOR courses) and Asia (postgraduate bedside teaching). He also cooperates with various US companies as tester of pre-market software/US programs or equipment/transducer, as well as for development of dedicated pediatric (3D/4DUS) probes, pediatric CT optimization, and so on*.

*Over the years Prof. Riccabona has been editor of six books and author of more than 50 chapters in various pediatric, ultrasound, pediatric radiology and radiology textbooks, not to forget his over 200 scientific publications – including multiple invited articles and review papers, numerous abstracts and course syllabus contributions, co-authorships and the like (over 1000)*.

*Prof. Riccabona received various Poster and Presentation prices and awards, the award for Eminent scientist 2007 and the Styrian Congress Award 2016 (for the ESPR annual meeting & postgraduate course 2015 in Graz)*.

